# Genome Sequence of *Corynebacterium glutamicum* Phage MicyPS

**DOI:** 10.17912/micropub.biology.001936

**Published:** 2026-02-03

**Authors:** Ombeline Rossier, Florence Constantinesco-Becker, Anne Lopes, Daniel Delaruelle, Ana A Arteni, Lydia Hassissene, Malika Ouldali, Laura Pieri, Avril Zappini, Katia Zaidi, Armand Tomasella, Perla Tannous, Michel Nouhra, Mickael Marques, Cindy Goodur, Louis Gachot, Erine Dumond, Alizée Dias, Heather Desolle, Meissan Chikhi, Chahine Belhachem, Adel Amriche, Christophe Regeard

**Affiliations:** 1 Université Paris-Saclay, CEA, CNRS, Institute for Integrative Biology of the Cell (I2BC), Gif-sur-Yvette, France; 2 Faculté des Sciences d’Orsay, Université Paris-Saclay, Orsay, France; 3 Ecole Universitaire de Premier Cycle, Faculté des Sciences d’Orsay, Université Paris-Saclay, Orsay, France

## Abstract

MicyPS is a bacteriophage with siphovirus morphology infecting *Corynebacterium glutamicum* strain MB001. It was isolated from soil near a henhouse in Villiers-sur-Marne, France. Its 78,208-bp genome encodes 115 predicted protein-encoding genes and 5 tRNAs. Based on gene-content similarity with actinobacteriophage PSonyx, MicyPS was assigned to the new cluster EQ.

**Figure 1. Plaque and virion morphology of phage MicyPS:**
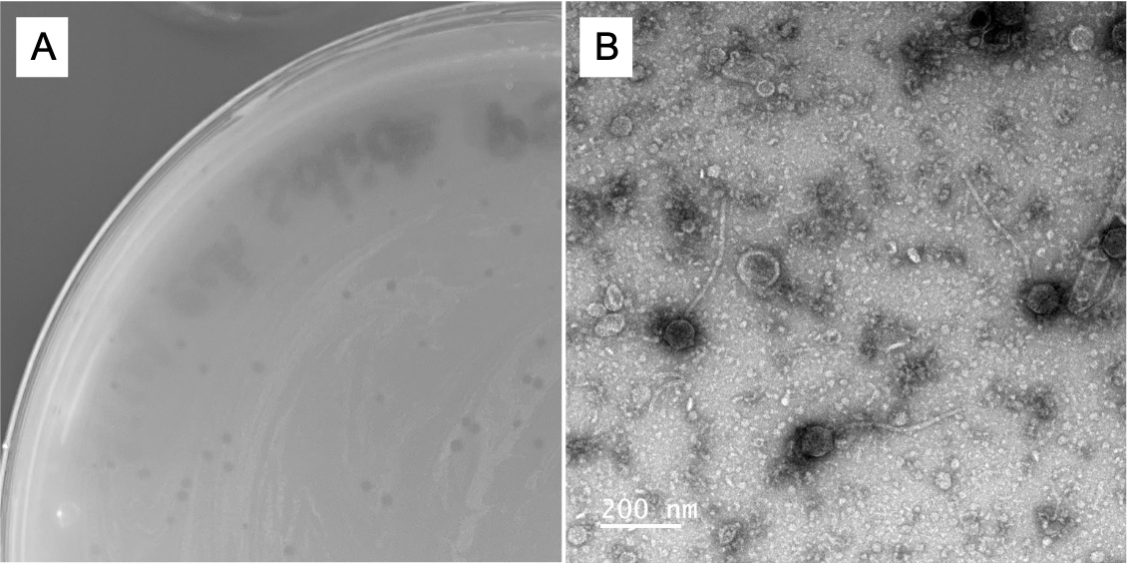
**(A)** Clear plaques observed in the LB agar overlay supplemented with 1 mM CaCl _2_ and *C* . *glutamicum* strain MB001. A portion of a standard 100-mm Petri dish is shown. **(B)** Negative staining of MicyPS phages with 2% w/v uranyl acetate observed with a JEOL1400 microscope operated at 80 kV and equipped with RIO9 direct electron detection camera (Ametek/Gatan). The magnification used was 12,000x with a pixel size of 0.56 nm at the level of the specimen. Bar, 200 nm.

## Description

Despite the industrial significance of *Corynebacterium glutamicum* in the production of glutamate and lysine, only seven bacteriophages infecting this species have been fully characterized to date with respect to both genome sequence and virion morphology (Moreau et al. 1995; Bukovska et al. 2006; Chen et al. 2008; Lobanova et al. 2017; Yomantas et al. 2018; Hünnefeld et al. 2021; Rossier et al. 2024). This limited number underlines an important gap in our understanding of phage diversity, biology, and their potential impact on fermentation processes involving *C.* *glutamicum* .

Phage MicyPS was isolated from soil near a henhouse in Villiers-sur-Marne, France (GPS 48.829132 N, 2.537859 E). Soil was incubated for 1 hour with 25 mL LB broth (5 g/L NaCl; 5 g/L yeast extract; 10 g/L tryptone), whilst shaking at 30° C. Following centrifugation at 4000 xg for 15 minutes, the supernatant was passed through a 0.45-μm filter and supplemented with 1 mM CaCl _2_ and inoculated with *C* . *glutamicum* strain MB001, a derivative of the reference strain ATCC 13032 in which the prophages CGP1, CGP2, and CGP3 were deleted (Hünnefeld et al. 2021). After shaking for 24 hours at 30° C, enrichment cultures were centrifuged and the supernatant was filtered through a 0.22-μm filter. Five mL of the supernatant were then spotted on double agar plate supplemented with 1 mM CaCl _2_ and strain MB001. After 24 hours of incubation at 30° C, individual plaques were visible and further purified through three rounds of single plaque picking and plating. Plaques were clear and between 0.5 and 3-mm in diameter ( Figure 1A ). Negative-staining electron microscopy revealed a siphovirus morphology with capsids 75 (±4) nm in diameter and tails 320 (±26) nm long (n=20).

Viral DNA was extracted using the PCI/SDS protocol (see Methods). NEB Ultra II FS Kit was used to prepare the library that was sequenced using Illumina NextSeq 1000, yielding 1,078,713 single-end 100-base reads. Raw reads were trimmed with cutadapt 4.7 (using the option: –nextseq-trim 30) and filtered with Skewer 0.2.2 (using the options: -q 20 -Q 30 -n -l 50) prior to assembly (Martin 2011; Jiang et al. 2014). De novo assembly was performed with Newbler v2.9 as previously described (Russell 2018) and further checked with Consed v.29 and Unicycler (Gordon et al. 1998; Wick et al. 2017). The resulting genome was 78,208-bp in length, with a G+C content of 53.2% and circularly permutated ends. Based on gene content similarity of 76.2% with the previously singleton phage PSonyx (Rossier et al. 2024), phage MicyPS was assigned to the new cluster EQ in the Actinophage database PhagesDB (Russell and Hatfull 2017).

Genome annotation identified 120 putative genes including 5 tRNAs with the following tools: DNA master v5.23.6 (Pope and Jacobs-Sera 2018) which incorporates Glimmer v3.02 (Delcher et al. 2007) and GeneMark v2.5p (Besemer and Borodovsky 2005), Aragorn v1.2.40 (Laslett 2004), tRNAscan-SE v2.0 (Chan et al. 2021) and Phamerator (Cresawn et al. 2011). Start sites were selected based on gene length, minimal gaps or overlaps, RBS scores, and BLASTP alignment. Functional annotation was performed with BLASTP (Altschul et al. 1990) using PhagesDB and NCBI non-redundant databases (e values < 0.001) and with HHpred (Soding et al. 2005) using databases PDB_mmCIF70_30_Mar, Pfam-A_v37, Uniprot-Swissprot-viral70_3_Nov_2021, and NCBIConservedDomain(CD)_v3.19. Using DeepTMHMM v1.0.42 (Hallgren et al. 2022), nine proteins were predicted to localize to the membrane. Only 35% of putative genes had a predicted function. One region (representing 40% of the genome) is transcribed in the rightward direction and encodes proteins involved in virion assembly and host lysis. The next 40%, transcribed leftwards, contains tRNAs and genes likely involved in DNA replication. Located in this region, nine (of a total of 15) predicted genes have no significant similarity with any other predicted genes in the Actinophage database. These genes are termed orphams. &nbsp;The final 20%, transcribed rightwards, end with a large putative gene which is predicted to encode a 2,873-amino-acid protein of unknown function. Both phages in cluster EQ (MicyPS and PSonyx) have no identifiable immunity repressor or integrase functions suggesting the use of the lytic cycle for replication.

**Nucleotide sequence accession numbers**

MicyPS is available at GenBank with Accession No. PV876931 and Sequence Read Archive (SRA) No. SRX28943171.

## Methods

Viral DNA was extracted using the PCI/SDS protocol ( https://phagesdb.org/media/workflow/protocols/pdfs/PCI_SDS_DNA_Extraction_2.2013.pdf ).

## References

[R1] Altschul Stephen F., Gish Warren, Miller Webb, Myers Eugene W., Lipman David J. (1990). Basic local alignment search tool. Journal of Molecular Biology.

[R2] Besemer J., Borodovsky M. (2005). GeneMark: web software for gene finding in prokaryotes, eukaryotes and viruses. Nucleic Acids Research.

[R3] Bukovska Gabriela, Klucar Lubos, Vlcek Cestmir, Adamovic Jan, Turna Jan, Timko Jozef (2006). Complete nucleotide sequence and genome analysis of bacteriophage BFK20 — A lytic phage of the industrial producer Brevibacterium flavum. Virology.

[R4] Chan Patricia P, Lin Brian Y, Mak Allysia J, Lowe Todd M (2021). tRNAscan-SE 2.0: improved detection and functional classification of transfer RNA genes. Nucleic Acids Research.

[R5] Chen Chang-Lin, Pan Tzu-Ying, Kan Shu-Chen, Kuan Yi-Chia, Hong Lian-Yu, Chiu Kun-Ruei, Sheu Ching-Sen, Yang Jui-Sen, Hsu Wen-Hwei, Hu Hui-Yu (2008). Genome sequence of the lytic bacteriophage P1201 from Corynebacterium glutamicum NCHU 87078: Evolutionary relationships to phages from Corynebacterineae. Virology.

[R6] Delcher Arthur L., Bratke Kirsten A., Powers Edwin C., Salzberg Steven L. (2007). Identifying bacterial genes and endosymbiont DNA with Glimmer. Bioinformatics.

[R7] Gordon David, Abajian Chris, Green Phil (1998). *Consed:*
A Graphical Tool for Sequence Finishing. Genome Research.

[R8] Hallgren Jeppe, Tsirigos Konstantinos D., Pedersen Mads Damgaard, Almagro Armenteros José Juan, Marcatili Paolo, Nielsen Henrik, Krogh Anders, Winther Ole (2022). DeepTMHMM predicts alpha and beta transmembrane proteins using deep neural networks.

[R9] Hünnefeld Max, Viets Ulrike, Sharma Vikas, Wirtz Astrid, Hardy Aël, Frunzke Julia (2021). Genome Sequence of the Bacteriophage CL31 and Interaction with the Host Strain Corynebacterium glutamicum ATCC 13032. Viruses.

[R10] Jiang Hongshan, Lei Rong, Ding Shou-Wei, Zhu Shuifang (2014). Skewer: a fast and accurate adapter trimmer for next-generation sequencing paired-end reads. BMC Bioinformatics.

[R11] Laslett D. (2004). ARAGORN, a program to detect tRNA genes and tmRNA genes in nucleotide sequences. Nucleic Acids Research.

[R12] Lobanova Juliya S., Gak Evgueni R., Andreeva Irina G., Rybak Konstantin V., Krylov Alexander A., Mashko Sergey V. (2017). Complete nucleotide sequence and annotation of the temperate corynephage ϕ16 genome. Archives of Virology.

[R13] Martin Marcel (2011). Cutadapt removes adapter sequences from high-throughput sequencing reads. EMBnet.journal.

[R14] Moreau S., Leret V., Le Marrec C., Varangot H., Ayache m., Bonnassie S., Blanco C., Trautwetter A. (1995). Prophage distribution in coryneform bacteria. Research in Microbiology.

[R15] Pope Welkin H., Jacobs-Sera Deborah (2017). Annotation of Bacteriophage Genome Sequences Using DNA Master: An Overview. Methods in Molecular Biology.

[R16] Rossier Ombeline, Labarre Cécile, Lopes Anne, Auberdiac Monique, Tambosco Kevin, Delaruelle Daniel, Abes Hakima, Arteni Ana A., Ouldali Malika, Pieri Laura, Afgoun Ryan, Anacleto Leonor, Beaure Nathan, Beghdad Meyssa, Bellom Nolwenn, Ben Hamou-Kuijpers Elsa, Boukamel Aïda, Carron James, Carta Vincent, Castelneau Lauriane, Chadaillac Zoe, Chaouat Elsa, Desmat Soline, Favel Keylian, Gabillot Eva, Gargar Melissa, Gautheret Madeleine, Gilles Esther, Lager Claire, Le Deit Amandine, Le vay Yoann, Lemercier Laure, Litvinov Anastassiya, Moussi Samir, Prevot Marion, Rehala Marion, Rodrigues Chloë, Sambe Ramatoulaye, Srimoorthy Ashvini, Tillay Tiroumagale M., Verhoeven Cerise, Vittaz Pauline, Wu Jacqueline, Regeard Christophe (2024). Genome sequence of PSonyx, a singleton bacteriophage infecting
*Corynebacterium glutamicum*. Microbiology Resource Announcements.

[R17] Russell Daniel A. (2017). Sequencing, Assembling, and Finishing Complete Bacteriophage Genomes. Methods in Molecular Biology.

[R18] Russell Daniel A, Hatfull Graham F (2016). PhagesDB: the actinobacteriophage database. Bioinformatics.

[R19] Soding J., Biegert A., Lupas A. N. (2005). The HHpred interactive server for protein homology detection and structure prediction. Nucleic Acids Research.

[R20] Wick Ryan R., Judd Louise M., Gorrie Claire L., Holt Kathryn E. (2017). Unicycler: Resolving bacterial genome assemblies from short and long sequencing reads. PLOS Computational Biology.

[R21] Yomantas Yurgis A. V., Abalakina Elena G., Lobanova Juliya S., Mamontov Victor A., Stoynova Nataliya V., Mashko Sergey V. (2018). Complete nucleotide sequences and annotations of φ673 and φ674, two newly characterised lytic phages of Corynebacterium glutamicum ATCC 13032. Archives of Virology.

